# Impact of the COVID-19 Pandemic on Takotsubo Syndrome: Updated Insights From a Retrospective Analysis Using the Nationwide Inpatient Sample

**DOI:** 10.7759/cureus.73546

**Published:** 2024-11-12

**Authors:** Viet Nghi Tran, Chau Doan Nguyen, Hong Hieu Truong, Hoang Nhat Pham, Amreen Dhindsa, Thach Nguyen Ngoc, Phillip Tran

**Affiliations:** 1 Internal Medicine, Weiss Memorial Hospital, Chicago, USA; 2 Medicine, Texas Tech University Health Sciences Center, Amarillo, USA; 3 Medicine, Ascension Saint Francis, Evanston, USA; 4 Medicine, University of Milan, Milan, ITA; 5 Cardiology, Methodist Hospital Merrillville, Merrillville, USA; 6 Cardiology, Nam Can Tho University, Can Tho, VNM

**Keywords:** covid-19, global crisis, stress-related cardiomyopathy, takotsubo cardiomyopathy, takotsubo syndrome, world pandemic

## Abstract

Background

We explored the impact of the COVID-19 pandemic on the prevalence and outcomes of takotsubo syndrome (takotsubo) using the National Inpatient Sample (NIS) to compare trends before and during the pandemic.

Methods

This retrospective study examined data from over 137 million admissions during 2017-2018 (pre-pandemic) and 2020-2021 (pandemic).

Results

Our analysis revealed a marked increase in takotsubo prevalence, from 109.07 to 131.19 per 100,000 admissions over each two-year period. The majority of patients were elderly females, with a notable rise in associated comorbidities such as hypertension and diabetes during the pandemic. Mortality among takotsubo patients rose significantly, with an adjusted odds ratio of 1.28. Patients with concurrent COVID-19 infection had a mortality rate of 23.02%, significantly higher than 7.61% for those without. The length of hospital stays also increased, particularly among COVID-19 patients. Interestingly, the odds of developing takotsubo were lower in COVID-19 patients than in non-infected individuals, suggesting a broader pandemic-related stress impact.

Conclusion

Our findings highlight a complex interaction between viral infections, stress, and cardiovascular health, underscoring the need for integrated care strategies during global health crises.

## Introduction

Takotsubo syndrome (takotsubo), or stress-induced cardiomyopathy, was first reported in 1990 by Sato and is well-recognized for being triggered by both physical and emotional stress [1,2]. This condition has also been reported to be directly related to viral infections such as influenza, parvovirus, and the recent COVID-19 [3-8]. However, the COVID-19 virus brought an unpredictable global health crisis, significantly increasing stress worldwide, which likely had an indirect impact on the prevalence of takotsubo syndrome. This association presents an exceptional opportunity to examine both the direct and indirect effects of a pandemic-created virus on stress-induced cardiomyopathy, and more broadly, stress-related cardiac incidents. Utilizing the latest data from the National Inpatient Sample (NIS), this study investigates changes in the prevalence, clinical outcomes, and hospital length of stay for patients with takotsubo during the pandemic, compared to pre-pandemic periods. Moreover, it explores the mortality odds between takotsubo patients with COVID-19 infection and those without, providing the most updated insights into the intricate dynamics between the viral pandemic and takotsubo syndrome.

## Materials and methods

Study design and data source

We conducted a retrospective study utilizing the NIS database to investigate the impact of the COVID-19 pandemic on takotsubo syndrome. The NIS, part of the Healthcare Cost and Utilization Project by the Agency for Healthcare Research and Quality, is the largest publicly available all-payer inpatient healthcare database in the United States, covering 97% of the population [9]. It provides comprehensive information on inpatient stays, including patient demographics, diagnoses, procedures, and hospital characteristics. The NIS approximates a 20% stratified sample of discharges from US community hospitals, excluding rehabilitation and long-term acute care hospitals [10]. We analyzed data from two periods: pre-pandemic (2017-2018) and during the pandemic (2020-2021).

Study population

The study included all recorded admissions with a diagnosis of takotsubo syndrome, identified using the International Classification of Diseases, Tenth Revision, Clinical Modification (ICD-10-CM) codes. The dataset included a total of 137,015,481 admissions, with 71,325,934 admissions occurring pre-pandemic (2017-2018) and 65,689,547 during the pandemic (2020-2021). We applied sampling weights provided by the NIS to ensure that the estimates were representative of the national inpatient population.

Variables

The primary variable was the prevalence of takotsubo syndrome, analyzed across two distinct periods: pre-pandemic (2017-2018) and pandemic (2020-2021). Key demographic factors, including age, gender, and race, were examined to identify trends in the two populations. Clinical variables included comorbidities such as hypertension, diabetes, hyperlipidemia, and chronic kidney disease, along with the Charlson Comorbidity Index to assess the overall comorbidity burden. Outcome variables were mortality during hospitalization and length of hospital stay. Additionally, hospital-related factors such as region, bed size, and teaching status were also included as covariates in the regression models.

Statistical analysis

Descriptive statistics summarized patient demographics and baseline clinical characteristics. Differences between pre-pandemic and pandemic periods were compared using Pearson chi-squared tests for categorical variables and descriptive statistics or t-tests for continuous variables. Logistic regression models were employed to assess the odds of mortality, adjusting for potential confounders and covariates, including COVID-19 status, age, gender, race, income, comorbidity (hypertension, diabetes, hyperlipidemia, chronic kidney disease), Charlson Comorbidity Index, and hospital-related factors. Linear regression analyses were used to investigate the impact of the pandemic and COVID-19 status on the length of stay, adjusting for similar covariates. Additionally, we also calculated the mortality odds between takotsubo admissions with versus without COVID-19 infection during the pandemic (2020-2021) by using logistic regression analysis. P-value <0.05 indicates statistical significance. All analyses were performed using STATA version 17.0 (StataCorp LLC, College Station, TX), incorporating complex survey design features into the analysis to reflect the stratified sampling methodology of the NIS [10,11].

Ethical considerations

Given the de-identified nature of the NIS data, institutional review board approval and individual patient consent were not required.

## Results

**Figure 1:**
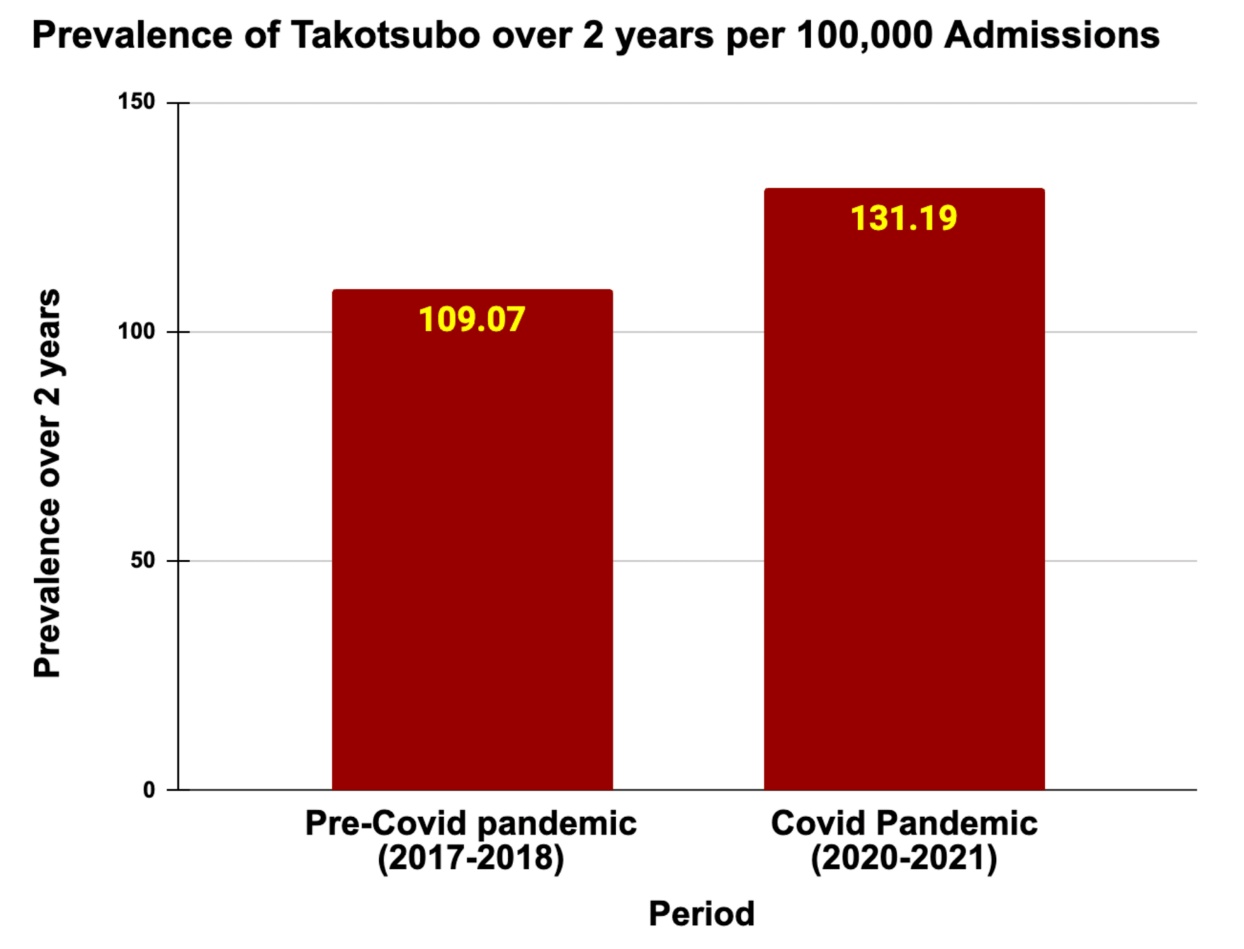
Two-year prevalence of takotsubo prior versus during the COVID-19 pandemic.

The two-year prevalence of takotsubo increased significantly from 109.07 per 100,000 admissions in the pre-pandemic period of 2017-2018 to 131.19 per 100,000 admissions during the pandemic in 2020-2021 (P < 0.001) (Table 1 and Figure 1). The average age of takotsubo patients remained steady at approximately 67 years. Notably, the majority of takotsubo patients were female, comprising 83.47% pre-pandemic and 80.30% during the pandemic. Racial distribution displayed slight changes, with the proportion of White patients decreasing from 80.22% to 78.96%, but not statistically significant (P = 0.26). The prevalence of common comorbidities rose during the pandemic, with hypertension increasing from 31.98% to 33.75% (P = 0.002), diabetes from 23.88% to 24.94% (P = 0.0314), chronic kidney disease from 13.34% to 14.59% (P = 0.0008), and hyperlipidemia from 44.72% to 47.29% (P = 0.0001). Admissions with a Charlson Comorbidity Score greater than two also went up to 46.19% during the pandemic compared to 42.64% in the prior period (P < 0.001). These baseline characteristics are included in Table 2.

**Table 1:** Two-year prevalence of takotsubo syndrome in the pre-pandemic (2017-2018) versus in the COVID-19 pandemic (2020-2021).

Period	Total admissions	Takotsubo cases	Prevalence over 2 years per 100,000 admissions
Pre-COVID-19 pandemic (2017-2018)	71,325,934	77,795	109.07
COVID-19 pandemic (2020-2021)	65,689,547	86,175	131.19
Total	137,015,481	163,970	119.67

**Table 2:** Baseline characteristics of patients with takotsubo between the pre-COVID-19 pandemic versus the COVID-19 pandemic. ^P^: Pearson chi-square test; CKD: chronic kidney disease; ESRD: end-stage renal disease. P-value < 0.05 indicates statistical significance.

	Pre-COVID-19 pandemic (2017-2018)	COVID-19 pandemic (2020-2021)	P-value
Age	66.9 (95% CI: 66.6-67.2)	66.7 (95% CI: 66.5-67.0)	-
Female	83.47%	80.30%	<0.001^P^
Race			0.259^P^
White	80.22%	78.96%	-
Black	8.08%	8.92%	-
Hispanic	6.61%	6.63%	-
Asian or Pacific Islander	2.08%	2.39%	-
Native American	0.68%	0.65%	-
Other	2.32%	2.45%	-
Hypertension	31.98%	33.75%	0.002^P^
Diabetes	23.88%	24.94%	0.031^P^
Hyperlipidemia	44.72%	47.29%	<0.001^P^
CKD stage			0.0008^P^
No CKD	86.66%	85.41%	-
Unspecific CKD	3.32%	3.07%	-
Stage 3 CKD	6.73%	7.91%	-
Stage 4 CKD	1.25%	1.46%	-
Stage 5 CKD	0.12%	0.17%	-
ESRD	1.93%	1.99%	-
Charlson Comorbidity Score			<0.001^P^
Score ≤ 2	57.36%	53.81%	-
Score ≥3	42.64%	46.19%	-

**Figure 2:**
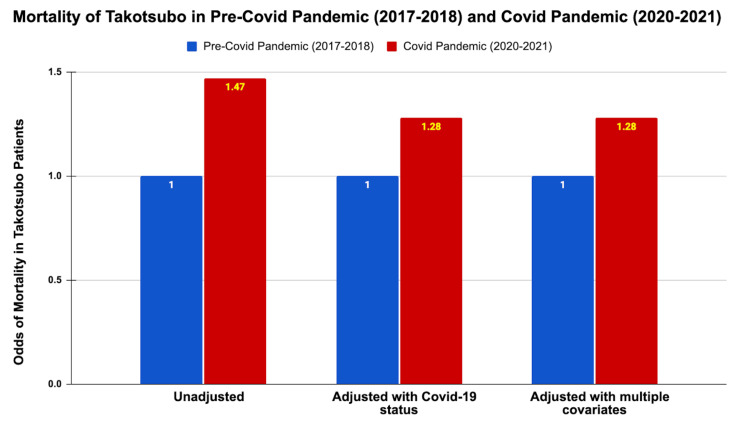
Mortality of takotsubo in the pre-COVID-19 pandemic (2017-2018) and during the COVID-19 pandemic (2020-2021).

**Figure 3:**
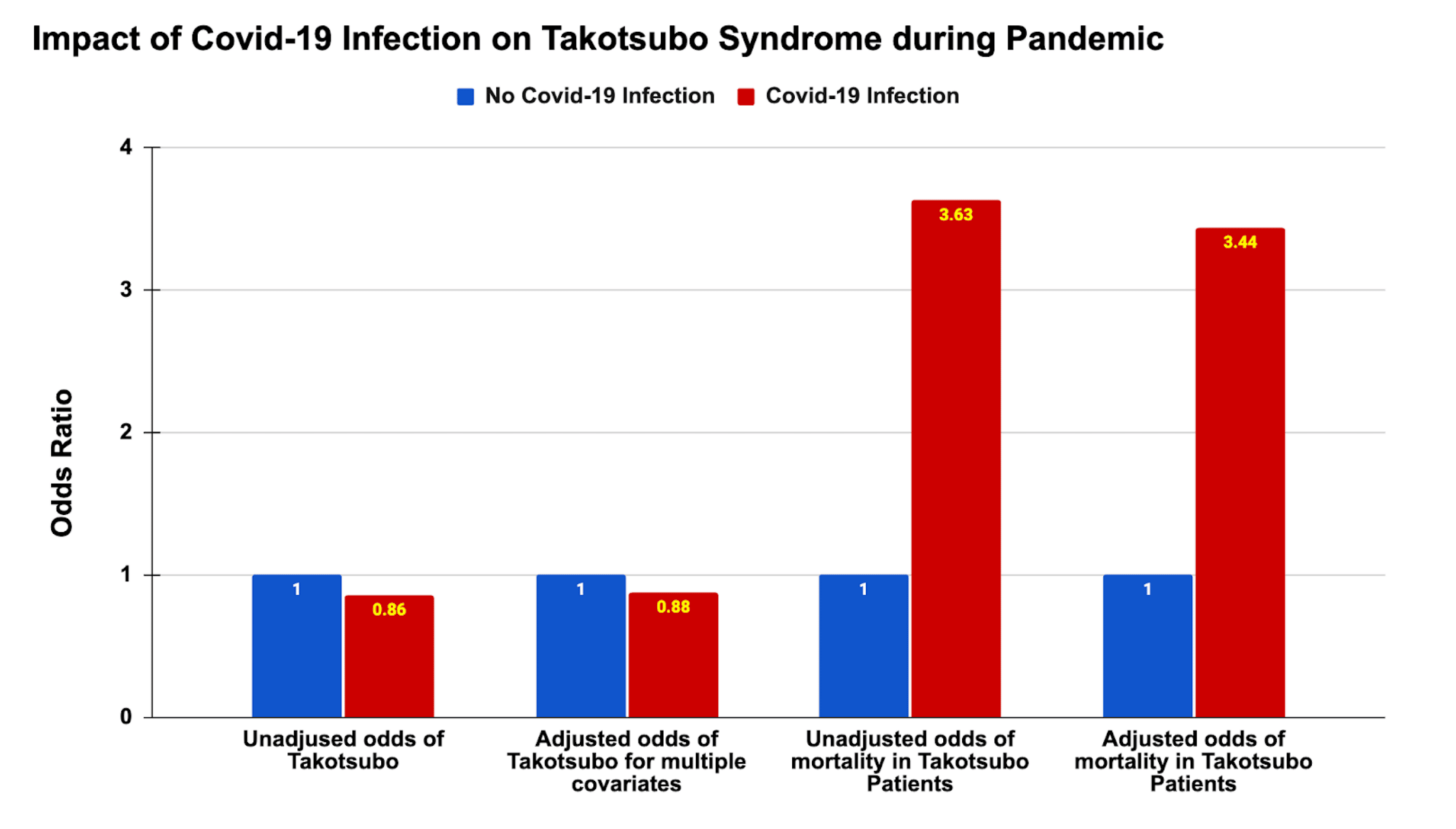
Impact of COVID-19 infection on takotsubo syndrome during the pandemic in 2020-2021.

Mortality rates among takotsubo patients were notably higher during the pandemic, with an unadjusted odds ratio (OR) of 1.47 (95% CI: 1.35-1.61, P < 0.001). Even after adjusting for COVID-19 status, the odds of mortality remained significantly elevated (adjusted OR: 1.28, 95% CI: 1.17-1.41, P < 0.001). Further adjustments for the baseline characteristics, income, the Charlson Comorbidity Index, and hospital-related factors confirmed the persistent increase in mortality odds during the pandemic (OR: 1.28, 95% CI: 1.17-1.41, P < 0.001) (Table 3 and Figure 2). Of note, during the pandemic, takotsubo patients with COVID-19 infection had a dramatically higher mortality rate of 23.02% compared to 7.61% in those without COVID-19 infection (P < 0.001) (Figure 3).

**Table 3:** Impact of the COVID-19 pandemic on takotsubo syndrome. ^P^: Pearson chi-square test; ^L^: logistic regression; ^Ln^: linear regression. P-value < 0.05 indicates statistical significance.

	Pre-COVID-19 pandemic (2017-2018)	COVID-19 pandemic (2020-2021)	95% confidence interval	P-value
Mortality rate in takotsubo patients (%)	6.04	8.67	-	<0.001^P^
Odds of mortality in takotsubo patients				
-Unadjusted	1	1.47	1.35 - 1.61	<0.001^L^
-Adjusted with COVID-19 status	1	1.28	1.17 - 1.41	<0.001^L^
-Adjusted with multiple potential confounders and covariates	1	1.28	1.17 - 1.41	<0.001^L^
Length of stay (LOS) of takotsubo admissions				
-Mean (days) (95% CI)	6.9 (6.7-7.1)	7.2 (7.1-7.5)	-	-
-Day increase in LOS	0	0.43	0.15 - 0.70	0.003^Ln^
-Adjusted with COVID-19 status (days)	0	0.12	-0.16 - 0.39	0.404^Ln^
-Adjusted with multiple potential confounders covariates (days)	0	-0.004	-0.24 - 0.23	0.975^Ln^

The length of stay for admissions with takotsubo increased from 6.90 days pre-pandemic to 7.32 days during the pandemic (Table 3). After adjusting for COVID-19 status, the effect of the pandemic period on length of stay became not statistically significant (P = 0.404). However, COVID-19 infection remained a strong predictor of increased length of stay, with an adjusted coefficient of 4.14 days (95% CI: 3.27-5.00, P < 0.001).

Interestingly, during the pandemic period, the odds of having takotsubo were significantly lower in patients with COVID-19 compared to those without, with an unadjusted odds ratio of 0.86 (95% CI: 0.81-0.92, P < 0.001). After adjusting for baseline characteristics, income, the Charlson Comorbidity Index, and hospital-related factors, patients with COVID-19 still had 11.20% lower odds of takotsubo (adjusted OR: 0.888) (Table 4 and Figure 3). Age and female gender were strong predictors of increased takotsubo risk, with each year of age associated with a slight increase in odds (OR: 1.026, 95% CI: 1.025-1.027, P < 0.001) and females having significantly higher odds of takotsubo compared to males (OR: 3.77, 95% CI: 3.61-3.94, P < 0.001).

**Table 4:** Effects of COVID-19 infection on takotsubo syndrome. ^P^: Pearson chi-square test; ^L^: logistic regression. P-value < 0.05 indicates statistical significance.

	No COVID-19 infection	COVID-19 infection	95% confidence interval	P-value
Prevalence rate of takotsubo	0.13	0.11	-	<0.001^P^
Odds of takotsubo				
-Unadjusted odds ratio	1	0.86	0.81 - 0.92	<0.001^L^
-Adjusted for multiple potential confounders and covariates	1	0.89	0.83 - 0.95	0.001^L^
Mortality in takotsubo patients				
-Mortality rate (%)	7.61	23.02	-	<0.001^P^
-Unadjusted odds of mortality	1	3.63	3.13 - 4.22	<0.001^L^
-Adjusted odds of mortality	1	3.44	2.91 - 4.07	<0.001^L^

## Discussion

The findings from our retrospective analysis reveal significant shifts in the epidemiology and outcomes of takotsubo syndrome during the COVID-19 pandemic, highlighting the intricate dynamics between the heightened stress levels and the viral infection itself, which have collectively affected certain aspects of takotsubo syndrome.

Takotsubo syndrome and stress

Many pathophysiologies have been proposed to explain why stress is a common trigger of takotsubo syndrome. The most widely accepted theory involves the release of catecholamines into the bloodstream during stress [12]. According to Lyon et al. and other researchers, high levels of circulating epinephrine during stress can cause myocardial stunning by altering intracellular signaling in ventricular cardiomyocytes from Gs to Gi protein via the β2-adrenoceptor. This switch protects against apoptosis induced by β1-adrenoceptors but has a negative inotropic effect, particularly in the apical myocardium, where β-adrenoceptor density is highest [13-17]. This phenomenon explains the typical apical ballooning observed on cardiac imaging in takotsubo patients [18,19].

Increased prevalence of takotsubo syndrome during the COVID-19 pandemic

Our study observed a notable increase in the two-year prevalence of takotsubo during the pandemic, rising from 109.07 per 100,000 admissions in the pre-pandemic period to 131.19 per 100,000 admissions during the pandemic. This increase may be attributed to the heightened levels of emotional and physical stress experienced globally during the pandemic [20]. Factors such as widespread fear, social isolation, financial insecurity, and the direct impact of COVID-19 infection likely contributed to this surge in takotsubo cases.

This trend aligns with other studies that have reported increased incidence rates of stress-induced cardiomyopathy during times of widespread stress, trauma, and natural disasters​​ [21-24]. The pandemic created a perfect storm of stressors, not only affecting individuals directly infected with the virus but also impacting the mental health of the general population.

Demographic and comorbidity patterns

The demographic profile of takotsubo patients remained relatively stable, with an average age of around 67 years and a predominance of female patients, which is consistent with the existing literature on takotsubo​​ [25,26]. However, the pandemic period saw a slight but significant rise in the prevalence of common comorbidities such as hypertension, diabetes, chronic kidney disease, and hyperlipidemia (Table 2). This increase could be reflective of the exacerbation of chronic conditions due to pandemic-related delays in routine healthcare and increased stress levels​​ [27].

Mortality and hospital stay

Noteworthy, there was a significant increase in mortality rates among takotsubo patients during the pandemic. The unadjusted odds ratio for mortality during the pandemic was 1.47, which remained elevated even after adjusting for covariates and confounding variables (adjusted OR: 1.28). This increase underscores the adverse impact of the pandemic on patients with takotsubo.

Furthermore, takotsubo patients with COVID-19 infection had considerably higher mortality rates compared to those without COVID-19 infection (23.02% vs. 7.61%). This significant change aligns with data from a systematic review by Ghasemi et al., reporting that the in-hospital mortality rate of takotsubo patients was 33.3% with COVID-19 positivity compared with 2.3% in those without COVID-19 [28]. This finding highlights the compounded risk posed by the combination of takotsubo and COVID-19 infection, necessitating heightened vigilance and aggressive management strategies for these patients.

Additionally, the length of hospital stay for takotsubo patients increased during the pandemic, primarily due to COVID-19 infection, because no statistically significant changes in the length of stay were observed after adjusting for COVID-19 status. COVID-19 infection was a strong predictor of prolonged hospitalization, adding an average of 4.14 days. This extended stay likely results from the need for more intensive monitoring, isolation, and treatment for COVID-19 patients, who are at a higher risk of complications.

Lower odds of takotsubo syndrome in patients with COVID-19 infection

An unexpected finding was the lower odds of takotsubo among patients with COVID-19 infection compared to those without (adjusted OR: 0.888) during the pandemic. This could be attributed to various factors, including the possibility that there was excessive emotional stress affecting the general population, especially the families and friends of COVID-19 patients, rather than the COVID-19 patients themselves. Additionally, the healthcare delivery system, healthcare resource allocation, and diagnostic focus might have shifted during the pandemic, potentially influencing the diagnosis process​​ [29-31].

Implications for clinical practice

The significant increase in takotsubo syndrome during the COVID-19 pandemic reinforces the strong link between stress and takotsubo, necessitating vigilance for risk factors and early signs of takotsubo syndrome, especially during any global or local crises. Higher comorbidities and mortality rates highlight the need for aggressive management and integrated care plans for takotsubo and COVID-19. The lower odds of takotsubo in COVID-19 patients during the pandemic suggest that the stress of the pandemic also impacted the general population significantly. Physicians should focus on early intervention, particularly in older and female patients, to improve clinical outcomes. These findings stress the importance of tailored care strategies for managing takotsubo during global health crises.

Limitations

This study has several limitations. The retrospective design and use of the NIS database limit causal inference, as the data lack specificity on the cause of in-hospital mortality, making it unclear if deaths were due to takotsubo, COVID-19, or other conditions. The administrative origin of the data may introduce inaccuracies, and the US-based sample may limit generalizability to populations in other countries. Additionally, the absence of information on individual psychosocial stressors, which are critical in the pathogenesis of takotsubo syndrome, may limit the precision and applicability of the findings to broader contexts.

## Conclusions

The COVID-19 pandemic has significantly affected the prevalence and outcomes of takotsubo, highlighting the intricate interplay between heightened stress levels, COVID-19 infections, and takotsubo syndrome. Our study reveals a significant increase in takotsubo prevalence and higher mortality rates during the pandemic. The findings advocate the importance of early identification, proactive management, and psychological support for at-risk populations such as elderly females. Of note, extended hospital stays in takotsubo patients were mostly related to the COVID-19 infection itself rather than the effects of the pandemic situation. On the other hand, the pandemic situation had excessive emotional stress, affecting not only COVID-19 patients but also the general population without COVID-19, which increased the odds of having takotsubo in the general population. Ultimately, addressing both the direct and indirect effects of a viral-induced global health crisis on takotsubo syndrome is crucial for improving patient outcomes and preventing surges of takotsubo syndrome in future pandemics.
